# Long-term outcome of extensive mitral valve reconstruction with autologous pericardium and artificial chordae for treatment of destructive active infective endocarditis of the mitral valve

**DOI:** 10.1186/s13019-022-01851-5

**Published:** 2022-05-03

**Authors:** Kazuma Handa, Takafumi Masai, Toshihiro Ohata, Tomohiko Sakamoto, Toru Kuratani

**Affiliations:** Department of Cardiovascular Surgery, Sakurabashi Watanabe Hospital, 2-4-32 Umeda, Kitaku, Osaka 530-0001 Japan

**Keywords:** Active infective endocarditis, Mitral valve reconstruction, Autologous pericardium

## Abstract

**Objective:**

Mitral valve (MV) repair is a well-accepted surgical approach for infective endocarditis (IE). In our hospital, extensive MV reconstruction with fresh autologous pericardium (AP) and artificial chordae (AC) has been performed for patients with profoundly extensive and destructive IE in which valve reconstruction would be extremely challenging, especially in young patients to avoid mechanical valve replacement. Long-term outcome including the future performance of the newly created leaflet has not been established.

**Methods:**

Five patients (54 (38–60) years of age; 3 men, 2 women) underwent this procedure from January 2011 to April 2022. In all patients, preoperative cardiac function was good (left ventricular ejection fraction, 69 (66–75)). After complete debridement of the infective valve tissue, the MV was reconstructed with large, fresh, trimmed AP and AC.

**Results:**

The reconstructed leaflets were anterior in three patients and posterior in four, and AC were placed in four patients. All patients showed an uneventful postoperative course and were discharged to home 36 (28–42) days postoperatively after completion of intravenous antibiotic therapy. Pre-discharge echocardiography revealed no or trivial mitral regurgitation (MR) in all patients. The median follow-up period was 9.6 (6.0–10.4) years, and no patients developed recurrence of the IE. The latest echocardiography in four patients showed trivial/mild MR with good leaflet function. One patient developed recurrence of MR, 5 months postoperatively.

**Conclusions:**

The short- and long-term outcomes of this procedure might be acceptable. This procedure might be considered as an effective and valuable option, especially in young patients.

## Introduction

Mitral valve repair is a widely accepted surgical approach for treatment of infective endocarditis of the mitral valve [1]. However, mitral valve replacement is sometimes required for profoundly extensive and destructive active infective endocarditis of the mitral valve because of the difficulty of valve reconstruction. In our hospital, extensive mitral valve reconstruction with fresh autologous pericardium and artificial chordae has been performed in such cases. This technique is used especially in young patients to avoid mechanical valve replacement, which necessitates lifelong anticoagulation with a vitamin K antagonist to prevent stroke and systemic embolization. In this procedure, it is crucial to clarify the long-term surgical outcome because the durability and future performance of the mitral leaflet after extensive reconstruction using fresh autologous pericardium have not been established. We herein report the surgical results of this procedure with a particular focus on the long-term outcome.

## Subjects

### Materials and methods

We retrospectively analyzed the medical records of five patients (three men, two women; median age, 54 (38–60) years) who underwent extensive mitral valve reconstruction with fresh autologous pericardium and artificial chordae for treatment of destructive active infective endocarditis of the mitral valve from January 2011 to April 2022 at our hospital. The patients’ preoperative demographic and echocardiographic data are presented in Table 1. The mitral regurgitation was severe in four patients and moderate in one. All patients had good preoperative cardiac function (median left ventricular ejection fraction, 69 (66–75)) and a stable respiratory condition.

**Table 1:** Preoperative characteristics

Age (year)	54 (38–60)
Male, n (%)	3 (60%)
Body surface area (m^2^)	1.65 (1.53–1.74)
Atrial fibrillation, n (%)	0 (0%)
Hemodialysis, n (%)	0 (0%)
Hypertension, n (%)	0 (0%)
Diabetes mellitus, n (%)	0 (0%)
Dyslipidemia, n (%)	0 (0%)
Coronary artery disease, n (%)	0 (0%)
Cerebrovascular disease, n (%)	0 (0%)
Miocardial infarction, n (%)	0 (0%)
History of cardiac surgery, n (%)	0 (0%)
NYHA class	
I/II	0 (0%)
III	5 (100%)
IV	0 (0%)
Echocardiographic parameters	
LVDd (mm)	55 (50–56)
LVDs (mm)	30 (30–34)
Ejection fraction (%)	69 (66–75)
LAD (mm)	40 (31–40)
MR grade	
Moderate	1 (20%)
Severe	4 (80%)
AR ≧ moderate	0 (0%)
TR ≧ moderate	0 (0%)

NYHA: New York Heart Association functional classification, LVDd: left ventricular end-diastolic dimension, LVDs: left ventricular end-systolic dimension, LAD: left atrial diameter, MR: mitral valve regurgitation, AR: aortic valve regurgitation, TR: tricuspid valve regurgitation

### Surgical technique

**Fig. 1:**
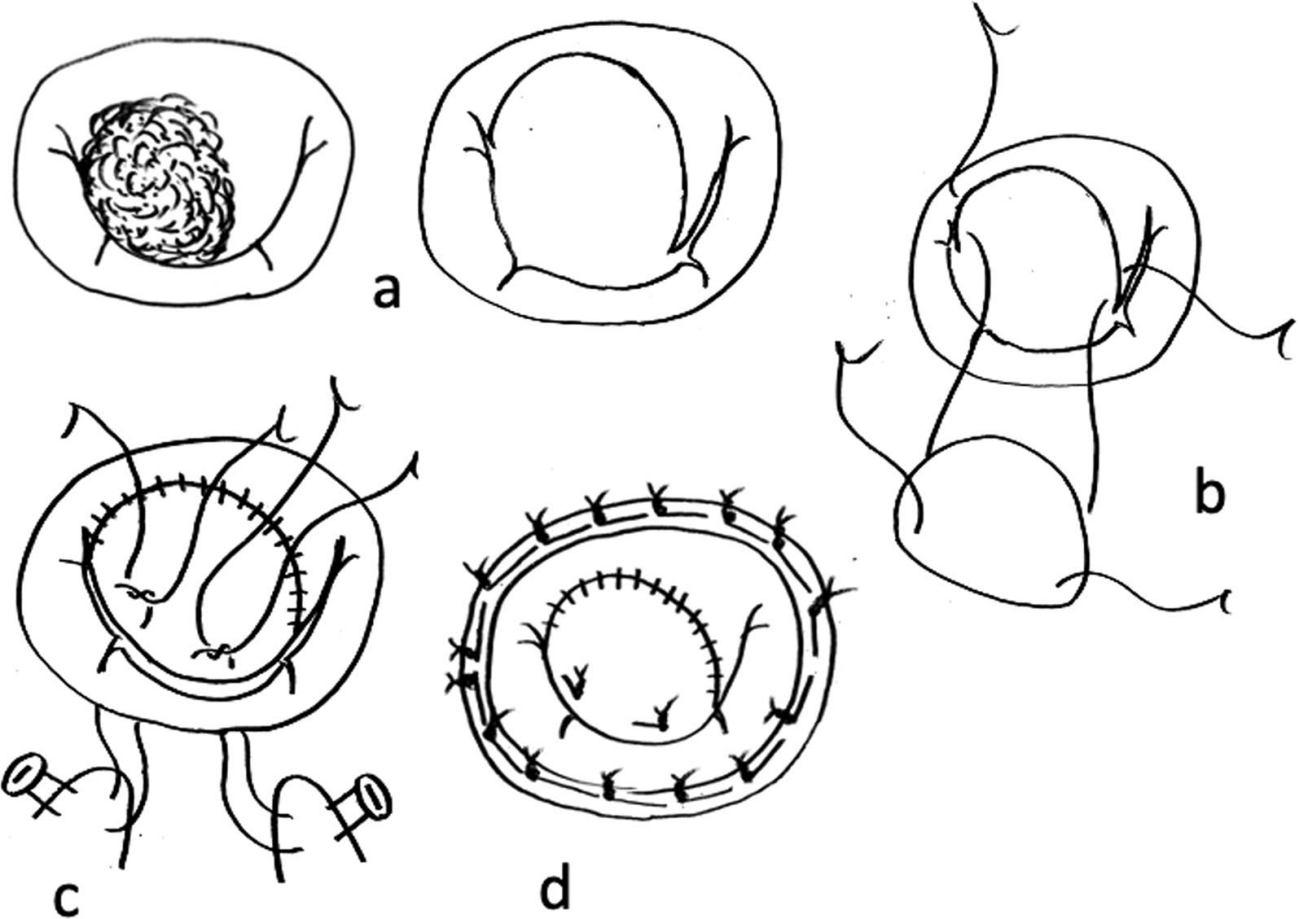
Surgical technique of mitral valve reconstruction with fresh autologous pericardium and artificial chordae. **a** Complete debridement of the infective and destructive valve tissue was performed first. **b** The fresh autologous pericardium was harvested and trimmed to an appropriate size corresponding to the defect of the mitral valve. The patch was attached to the annulus of the leaflet tissues. **c** Artificial chordae sutures were placed to the edge of the reconstructed pericardium. **d** Finally, mitral annuloplasty with an artificial annular ring was performed

Following median full sternotomy, cardiopulmonary bypass was routinely established by aortic and bicaval cannulation. As the intraoperative myocardial protection, initially, antegrade cold (15 °C) blood cardioplegic solution was administered, and then intermitted retrograde cold (15 °C) blood cardioplegic solution was administered every 20 min for maintenance of cardiac arrest. The mitral valve was exposed via a right-sided left atriotomy under cardioplegic arrest. Complete debridement of the infective and destructive valve tissue was performed first (Fig. 1a). Fresh autologous pericardium was harvested and trimmed to an appropriate size corresponding to the mitral valve defect. The size of the harvested pericardium was slightly larger than the size of the mitral valve defect, with a 5-mm-wide seam allowance. The mitral valve was then reconstructed with this fresh autologous pericardium. The base of the patch was attached to the mitral annulus with 4–0 polypropylene running sutures, and the side edges of the patch were sutured to the remnant of the leaflet tissues with 5–0 polypropylene sutures (Fig. 1b). As artificial chordae, 5–0 double-armed polytetrafluoroethylene sutures (Gore-Tex; W. L. Gore & Associates, Newark, DE, USA) were placed to the free edge of the reconstructed pericardium if needed (Fig. 1c). Finally, mitral annuloplasty with a prosthetic ring was performed (Fig. 1d). The prosthetic ring size was routinely determined according to the inter-trigonal distance and anterior leaflet height.

### Postoperative care and patient follow-up

Postoperative anticoagulation therapy with warfarin was continued only for the first 3 months. The prothrombin time–international normalized ratio was controlled at 1.5–2.5. Because no patients developed atrial fibrillation, subsequent continuation of anticoagulation therapy was not needed for any patients. Transthoracic echocardiograms were obtained before discharge and every year thereafter on an outpatient basis.

## Results

### Operative characteristics

**Fig. 2:**
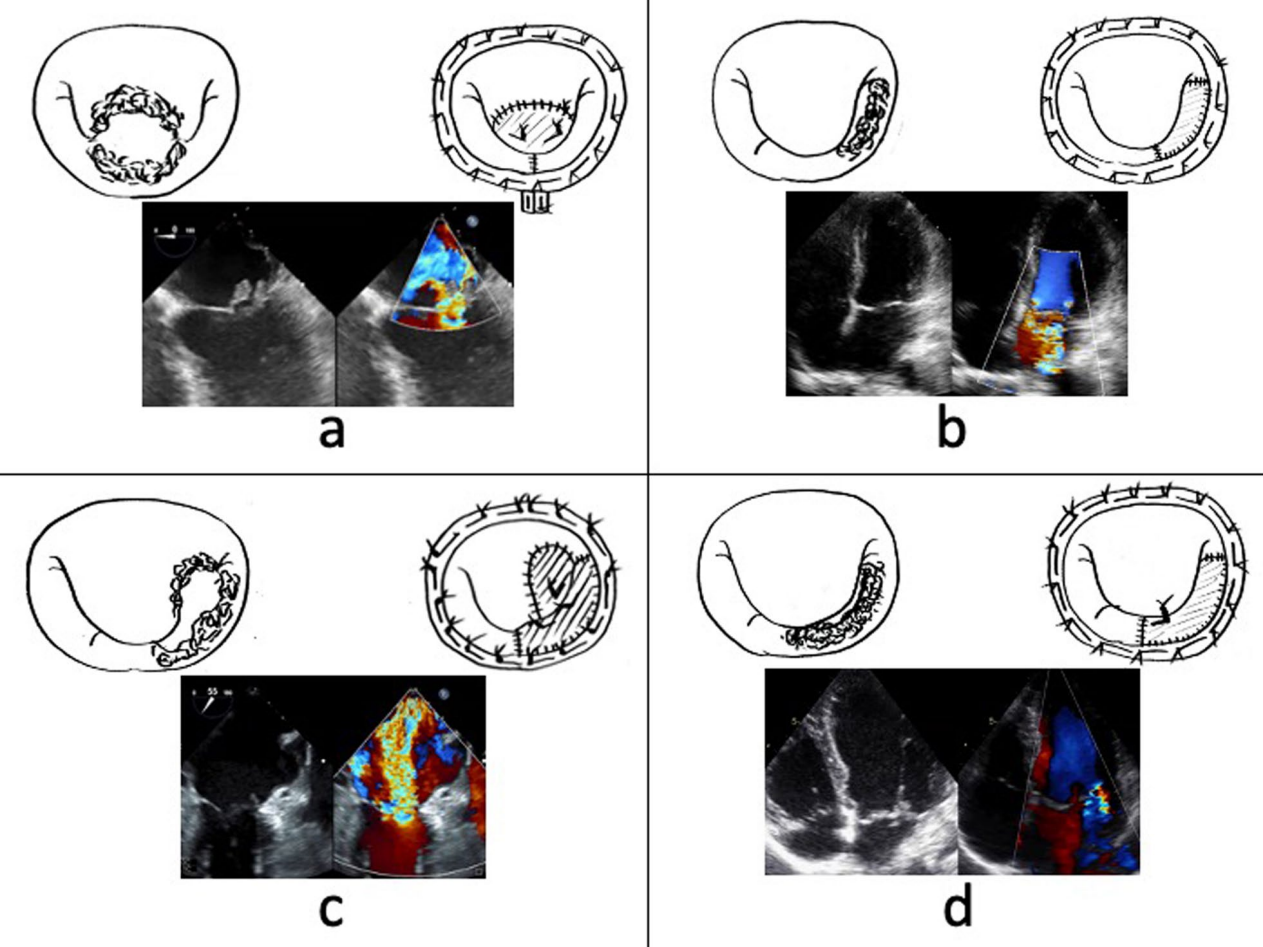
Surgical procedures for each patient with preoperative echocardiography. **a** Patient 1: A2 reconstruction with autologous pericardium + two pairs of artificial chordae + P2 quadrangular resection and suture + mitral annuloplasty using a 26-mm Physio II ring. **b** Patient 2: P3 reconstruction with autologous pericardium + mitral annuloplasty using a 28-mm Physio II ring. **c** Patient 3: A3/P2–3 reconstruction with autologous pericardium + three pairs of artificial chordae + mitral annuloplasty using a 26-mm Physio II ring. **d** Patient 4: P2–3 reconstruction with autologous pericardium + one pair of artificial chordae + mitral annuloplasty using a 32-mm Physio II ring

**Fig. 3:**
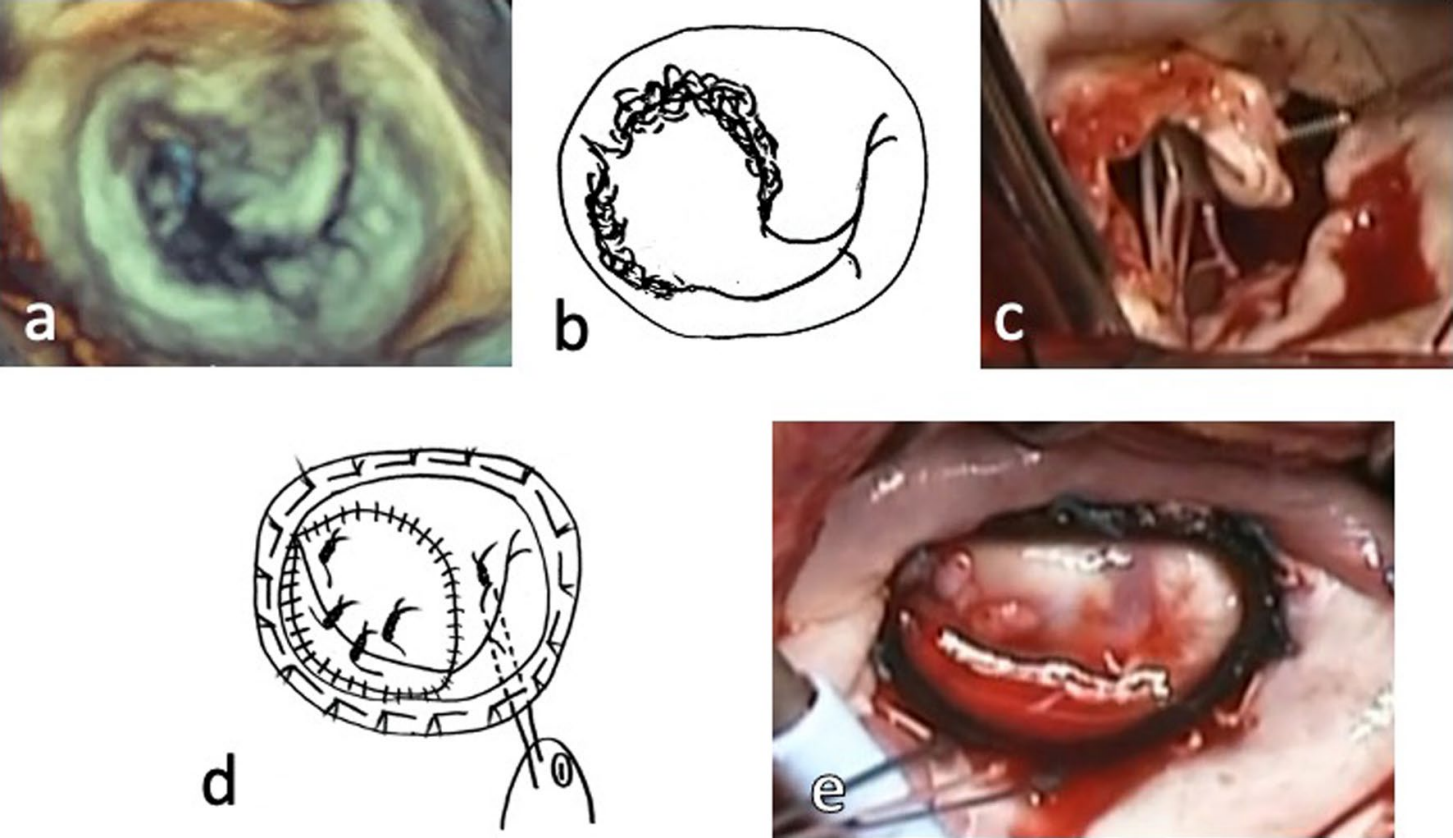
Operative findings and surgical procedure in Patient 5. **a** Preoperative real-time three-dimensional transesophageal echocardiography showed that the A1–2 and P1–2 segments of the mitral valve had been extensively destroyed. **b** The schema of the infective mitral valve. **c** Intraoperative finding of the infective mitral valve. **d** Surgical procedure: A1–2/P1–2 reconstruction with autologous pericardium + four pairs of artificial chordae + mitral annuloplasty using a 28-mm Memo 3D ring. **e** Intraoperative finding of the newly reconstructed mitral valve

The patients’ intraoperative characteristics are summarized in Table 2. In Patient 1, the A2 and P2 segments flailed with large vegetations. Blood culture showed methicillin-resistant coagulase-negative staphylococci. The patient’s mitral valve repair was performed by A2 leaflet reconstruction with autologous pericardium, two pairs of artificial chordae to the margin of the A2 pericardium, P2 quadrangular resection and suture, and mitral annuloplasty using a 26-mm Carpentier-Edwards Physio II ring (Edwards Lifesciences, Irvine, CA, USA) (Fig. 2a). Patient 2 had destructive infective endocarditis of the P3 segment caused by a viridans streptococcus. The P3 scallop was completely resected and reconstructed with autologous pericardium combined with mitral annuloplasty using a 28-mm Carpentier-Edwards Physio II ring (Fig. 2b). In Patient 3, A3 and P2–3 had been destroyed by a viridans streptococcus infection. We performed A3 leaflet and P2–3 scallop reconstruction with autologous pericardium, placement of three pairs of artificial chordae to each leaflet, and mitral annuloplasty using a 26-mm Carpentier-Edwards Physio II ring (Fig. 2c). Patient 4 had infective endocarditis in the P2–3 segment due to a viridans streptococcus infection. Surgery was performed with P2–3 scallop reconstruction with autologous pericardium, placement of one pair of artificial chordae to the P3 pericardium, and mitral annuloplasty using a 32-mm Carpentier-Edwards Physio II ring (Fig. 2d). In Patient 5, the A1–2 segment, anterior commissure leaflet, and P1–2 segment had been extensively destroyed by a methicillin-susceptible *Staphylococcus aureus* infection. The patient underwent A1–2 leaflet and P1–2 scallop reconstruction with autologous pericardium, placement of four pairs of artificial chordae to each pericardium, and mitral annuloplasty using a 28-mm Memo 3D ring (Sorin Biomedica Cardio S.r.L., Saluggia, Italy) (Fig. 3). No patients showed evidence of annular abscess formation.

**Table 2:** Operative characteristics of each patients

	Age Sex	Infective site (bacterial species)	Operative procedures
Patient 1	37 M	A2 + P2 (MRCNS)	A2 reconstruction with FAP + 2 pairs of AC + P2 quadrangular resection and suture + MAP (26 mm-PhysioII ring)
Patient 2	54 F	P3 (viridans streptococcus)	P3 reconstruction with FAP + MAP (28 mm-PhysioII ring)
Patient 3	63 M	A3 + P2-3 (viridans streptococcus)	A3 + P2-3 reconstruction with FAP + 4 pairs of AC + MAP (26 mm-PhysioII ring)
Patient 4	39 M	P2-3 (viridans streptococcus)	P2-3 reconstruction with FAP + 1 pairs of AC + MAP (32 mm-PhysioII ring)
Patient 5	57 F	A1-2 + AC + P1-2 (MSSA)	A1-2 + P1-2 reconstruction with FAP + 4 pairs of AC + MAP (28 mm-Mamo 3D)

M: male, F: female, FAP: fresh autologous pericardium, AC: artificial chordae, MAP: mitral annuloplasty, MRCNS: methicillin-resistant coagulase-negative staphylococci, MSSA: methicillin-susceptible staphylococcus aureus

### Perioperative outcomes

The early surgical results and pre-discharge echocardiographic data are shown in Table 3. All patients were discharged on foot. No patients developed complications such as stroke, wound infection, re-exploration for bleeding, or respiratory failure. No patients required a second run of cardiopulmonary bypass because of residual regurgitation. The median operative time was 251 (240–340) minutes, the median cardiopulmonary bypass time was 152 (129–213) minutes, and the median cross-clamp time was 130 (106–182) minutes. All patients had an uneventful postoperative course and were discharged to home 36 (28–42) days after the surgery with completion of intravenous antibiotic therapy. Pre-discharge echocardiography revealed no or trivial mitral regurgitation in all patients. No patients showed systolic anterior movement of the anterior leaflet. In all patients, postoperative left ventricular function was favorable (left ventricular end-diastolic / systolic diameter, 45 (44–46) mm; left ventricular ejection fraction, 64 (57–67) %).

**Table 3:** Early result and pre-discharge echocardiographic data

Operative time (min)	251 (240–340)
CPB time (min)	152 (129–213)
Cross clamp time (min)	130 (106–182)
Concomitant surgery	
PFO closure	1 (20%)
TAP, n (%)	0 (0%)
Maze, n (%)	0 (0%)
LAA closure, n (%)	0 (0%)
Second pump, n (%)	0 (0%)
Hospital stay (days)	36 (28–42)
Hospital mortality, n (%)	0 (0%)
Complications, n (%)	0 (0%)
Stroke, n (%)	0 (0%)
Heart failure, n (%)	0 (0%)
Atrial fibrillation, n (%)	0 (0%)
Infection, n (%)	0 (0%)
Bleeding, n (%)	0 (0%)
Embolic event, n (%)	0 (0%)
Renal failure, n (%)	0 (0%)
Tracheostomy, n (%)	0 (0%)
Echocardiographic parameters	
Pre-discharge TTE	
LVDd (mm)	45 (44–46)
LVDs (mm)	29 (28–32)
Ejection fraction (%)	64 (57–67)
LAD (mm)	36 (29–39)
MR	
None, n (%)	2 (40%)
Trivial n (%)	3 (60%)
≧ Moderate n (%)	0 (0%)
MV PHT	90 (75–94)
MV mPG	3.6 (2.7–5)
TR≧moderate	0 (0%)

CPB: cardiopulmonary bypass, PFO: patent foramen ovale, TAP: tricuspid annuloplasty, LAA: left atrial appendage, TTE: transthoracic echocardiography, PHT: pressure half time, LVDd: left ventricular end-diastolic dimension, LVDs: left ventricular end-systolic dimension, LAD: left atrial diameter, MR: mitral valve regurgitation, MV: mitral valve, mPG: mean pressure gradient,, TR: tricuspid valve regurgitation

### Long-term outcomes and latest echocardiographic data

**Fig. 4:**
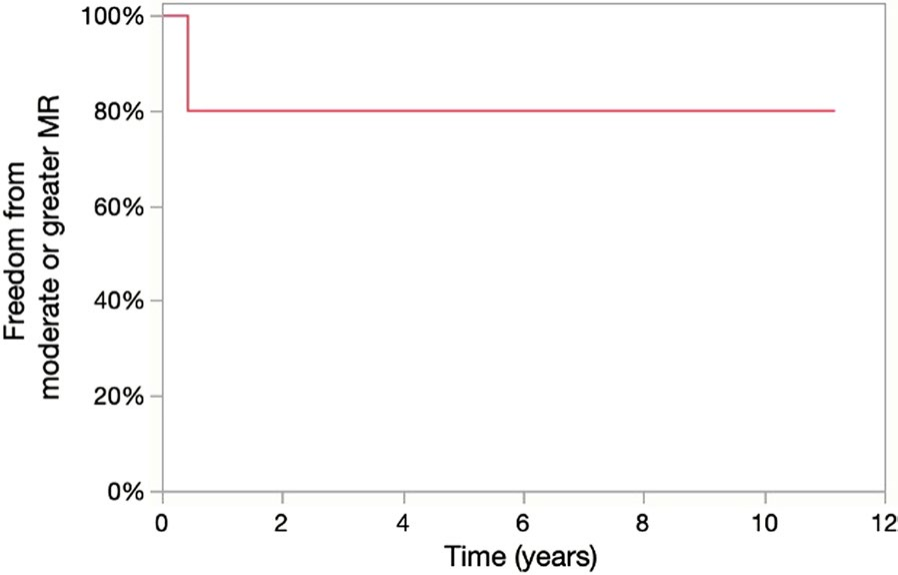
Long-term durability of this procedure; extensive mitral valve reconstruction with autologous pericardium and artificial chordae. MR: mitral valve regurgitation

The latest echocardiographic data of patients without recurrence are presented in Table 4. The median follow-up period was 9.6 (6.0–10.4) years. The average length of the latest echocardiographic follow-up was 9.6 (5.7–10.4) years. No patients developed recurrence of the infective endocarditis, and the inflammatory response as shown by the laboratory data remained negative after finishing antibiotic therapy. Long-term durability of this mitral procedure is represented by the Kaplan–Meier curve, which is shown in Fig. 4. In the latest echocardiographic examination, four patients had mitral regurgitation of less than 1 + with good cardiac function. The leaflet function of the autologous pericardium was good without any restricted movements, such as stiffness or calcification, in these four patients. Recurrence of severe mitral valve regurgitation was detected in Patient 3 at 5 months postoperatively and was probably due to detachment of the autologous pericardium suture line. This patient underwent mitral valve replacement with a bioprosthesis.

**Table 4:** Latest echocardiographic data of non-recurrent patients

LVDd (mm)	46 (41–48)
LVDs (mm)	28 (23–31)
Ejection fraction (%)	70 (63–74)
LAD (mm)	44 (24–44)
MR	
None, n (%)	0 (0%)
Trivial n (%)	2 (40%)
Mild n (%)	2 (40%)
MV mPG	3.5 (1.9–5)
TR ≧ moderate	0 (0%)

LVDd: left ventricular end-diastolic dimension, LVDs: left ventricular end-systolic dimension, LAD: left atrial diameter, MR: mitral valve regurgitation, MV: mitral valve, mPG: mean pressure gradient, TR: tricuspid valve regurgitation

## Discussion

Mitral valve repair has been increasingly utilized for the surgical treatment of active infective endocarditis because of its low early mortality rate and long-term outcomes exceeding those of mitral valve replacement [1]. However, mitral valve replacement is sometimes required for profoundly extensive and destructive active infective endocarditis of the mitral valve, for which mitral valve reconstruction is extremely challenging. Especially in young patients, mechanical mitral valve replacement is usually the standard procedure, after which lifelong anticoagulation with warfarin is mandatory to avoid stroke and systemic embolization. Some authors have reported extensive reconstruction of the mitral valve leaflets and chordae using autologous or bovine pericardium with or without chordal reconstruction for such devastating cases. These reports described only the short- or mid-term results; the long-term outcomes remain unclear because the durability of the pericardial leaflet is still controversial [2–5]. Furthermore, these reports included repair for chronic endocarditis, whereas repair for active endocarditis may be more challenging. Therefore, in this study, we aimed to clarify the long-term results of extensive mitral valve leaflet reconstruction with autologous pericardium focusing only on active infective endocarditis. Ito et al. [6] described 25 patients who underwent seamless reconstruction of the mitral leaflet and chordae with one piece of pericardium and demonstrated good short- and mid-term outcomes. In their observational study, only 6 of 25 patients had active endocarditis. The repaired lesion was the posterior leaflet together with its chordae (n = 3) and the commissural leaflet (n = 3). Miura, et al. [7] analyzed the relation of the localization and durability of the mitral valve repair in the 83 patients diagnosed with infective endocarditis. However, their study also included healed infective endocarditis (n = 17) and simple mitral valve repair (n = 66) without artificial chordae nor pericardium, and it was unclear about detail range of autologous pericardium patch repair whether the scallop was reconstructed with/without artificial chordae or not.

Therefore, there has been no study about the long-term result about only the extensive mitral valve scallop reconstruction with autologous pericardium for active infective endocarditis. In our study, all five patients had active endocarditis. The anterior leaflet was included with the repaired leaflet in three patients in whom large autologous pericardium and several artificial chordae were placed. Mitral regurgitation recurred in one of our five patients and similarly in one of six patients with active endocarditis in the study by Ito et al. [6] and Miura et al. [7] for the same reason (detachment of the pericardial suture line).

Another crucial issue is the durability of the autologous pericardium when implanted as part of the valve leaflet and whether the autologous pericardium should be treated by glutaraldehyde. Shomura et al. [8] reported good results with a mean follow-up of 4.5 years after mitral valve repair with glutaraldehyde-treated autologous pericardium in 139 patients, including 32 with active infective endocarditis (the 10-year rate of freedom from reoperation was 82%). Although the results for patients with only active infective endocarditis and the details of the surgical procedures (e.g., major or partial leaflet reconstruction and with or without chordal implantation) were not clear, this report demonstrated that the mid-term durability of glutaraldehyde-treated autologous pericardium might be favorable. Glutaraldehyde treatment may reportedly improve the durability of the reconstructed pericardium leaflet, providing a lower rate of calcification, shrinkage, or disruption than fresh autologous pericardium [9, 10]. In contrast, however, some reports have indicated that glutaraldehyde treatment might be associated with pericardial calcification [11, 12]. Excellent long-term outcomes of mitral valve leaflet repair using fresh autologous pericardium were recently reported (89% per 10 years of freedom from reoperation) [12]. This report demonstrated that glutaraldehyde treatment of the pericardium might be associated with late calcification and mitral valve stenosis due to leaflet thickening and loss of pliability. A conclusion has not yet been reached about the durability of the pericardium and how to manage the pericardium to obtain a good long-term result. In infective endocarditis with extensive valve destruction, multiple mitral valve scallops need to be reconstructed with a subvalvular device; polytetrafluoroethylene artificial chordae reconstruction is a widespread and familiar technique for many cardiac surgeons and good long-term results have been reported [14]. As reported by Ito et al. [6], reconstruction of the subvalvular apparatus with autologous pericardium seems to be a good and relatively simple method, but problems such as stiffening, shortening, and thickening of the autologous subvalvular apparatus may occur, and the long-term results are unknown and have not been compared to polytetrafluoroethylene. For these reasons, our surgical technique this procedure was to reconstruct only the valve leaflets with autologous pericardium, and the tendon cords were polytetrafluoroethylene artificial chordae.

A comparison of outcomes with bioprosthesis cannot be simply made. The median observation period in this study was 9.6 (5.7–10.4) years, but the number of patients was too small to compare with the durability of bioprosthesis; however, results of mitral bioprosthesis in patients younger than 70 years have been reported [13] and, as you say, a comparison with bioprosthesis would be very interesting. We would like to compare the durability of this procedure with that of bioprosthesis at the same age by further long-term observation.

To be sure, this technique is certainly not new. However, there are two new points in this study: (1) the study only included extensive autologous pericardial reconstruction of the mitral valve, and excluded patch reconstruction, as seen in other papers. Also, (2) the patients underwent only active infective endocarditis, excluding functional mitral regurgitation and healed infective endocarditis. In these two points, we believe that this paper may suggest new perspectives that this procedure might be an option for young patients with infective endocarditis with extensive valve destruction. Although the number of patients is certainly small, these are cases in which surgeons are struggling to make decisions in clinical practice, and we believe that this paper will be thought-provoking for many surgeons and physicians. Instead of immediate mitral valve replacement, this technique of mitral valve repair might be worth trying especially in young patients with profoundly extended and destructed active infective endocarditis.

## Limitations

The present study has some limitations. The median follow-up was only about 9.6 (6.0–10.4) years, the number of patients was limited, and this was an observational study at a single Japanese hospital without a control group.

## Conclusion

The long-term outcomes of mitral valve reconstruction with fresh autologous pericardium and artificial chordae for extensive and destructive active infective endocarditis of the mitral valve might be acceptable. This surgical procedure might be considered as an effective and valuable option, especially in young patients.

## Data Availability

The authors declare that all data in this article are available within this published article.

## References

[1] Ruttmann E, Legit C, Poelzl G (2005). Mitral valve repair provides improved outcome over replacement in active infective endocarditis. J Thorac Cardiovasc Surg.

[2] Araji OA, Barquero JM, Almendro M (2009). Replacement of A2 and A3 by pericardium due to endocarditis of the anterior leaflet of the mitral valve. Ann Thorac Surg.

[3] Nwaejike N, Ascinoe R (2010). Mitral valve repair for disruptive acute endocarditis: extensive replacement of posterior leaflet with bovine pericardium. J Card Surg.

[4] Ito T, Maekawa A, Sawaki S (2013). Reconstruction of mitral valve chordae and leaflets with one piece of autologous pericardium in extensively destructed mitral valve due to active infective endocarditis. Gen Thorac Cardiovasc Surg.

[5] Kimura S, Yamaki Y, Umesue M (2014). Reconstruction of anterior mitral leaflet using autologous pericardial patch combined with posterior leaflet sliding for active infective endocarditis. Gen Thorac Cardiovasc Surg.

[6] Ito T, Maekawa A, Aoki M (2014). Seamless reconstruction of mitral leaflet and chordae with one piece of pericardium. Eur J Cardiothorac Surg.

[7] Miura T, Obase K, Ariyoshi T (2020). Impact of lesion localozation on durability of mitral valve repair in infective endocarditis. Ann Thorac Surg.

[8] Shomura Y, Okada Y, Nasu M (2013). Late results of mitral valve repair with glutaraldehyde-treated autologous pericardium. Ann Thorac Surg.

[9] Ng CK, Nesser J, Punzengruber C (2001). Valvuloplasty with glutaraldehyde-treated autologous pericardium in patients with complex mitral valve pathology. Ann thorac Surg.

[10] Fukunaga N, Sakata R, Koyama T (2017). Reoperative analysis after mitral valve repair with glutaraldehyde-treated autologous pericardium. Interact Cardiovasc Thorac Surg.

[11] Liao K, Frater RW, LaPietra A (1995). Time-dependent effect of glutaraldehyde on the tendency to calcify of both autografts and xenografts. Ann Thorac Surg.

[12] Quinn RW, Wang L, Foster N (2020). Long-term performance of fresh autologous pericardium for mitral valve leaflet repair. Ann Thorac Surg.

[13] Jamieson WR, Gudas VM, Burr LH (2009). Mitral valve disease: if the mitral valve is not reparable/failed repair, is bioprosthesis suitable for replacement?. Eur J Cardiothorac Surg.

[14] Ibrahim M, Rao C, Savvopoulou M, Casula R, Athanasiou T (2014). Outcomes of mitral valve repair using artificial chordae. Eur J Cardiothorac Surg.

